# Development of an Ophthalmic Hydrogel to Deliver MG53 and Promote Corneal Wound Healing †

**DOI:** 10.3390/pharmaceutics17040526

**Published:** 2025-04-16

**Authors:** Heather L. Chandler, Sara Moradi, Spencer W. Green, Peng Chen, Christopher Madden, Luxi Zhang, Zhentao Zhang, Ki Ho Park, Jianjie Ma, Hua Zhu, Katelyn E. Swindle-Reilly

**Affiliations:** 1 College of Optometry, The Ohio State University, Columbus, OH 43210, USA; madden.80@osu.edu (C.M.); zhang.12677@osu.edu (L.Z.); 2 Department of Biomedical Engineering, The Ohio State University, Columbus, OH 43210, USA; smoradi.phd@gmail.com (S.M.); green.2317@osu.edu (S.W.G.); 3 Department of Surgery, The Ohio State University Wexner Medical Center, Columbus, OH 43210, USA; peng.chen@osumc.edu (P.C.); zhentao.zhang@osumc.edu (Z.Z.); hua.zhu@osumc.edu (H.Z.); 4 Division of Surgical Sciences, Department of Surgery, University of Virginia Medical School, Charlottesville, VA 22903, USA; kiho.park@virgina.edu (K.H.P.); jianjie.ma@virginia.edu (J.M.)

**Keywords:** corneal opacification, vascularization, MG53, thermosensitive hydrogel

## Abstract

**Background/Objective:** A clinical need exists for more effective therapeutics and sustained drug delivery systems to promote ocular surface healing. This study tested the hypothesis that a novel biodegradable, thermoresponsive hydrogel loaded with the human recombinant (rh)MG53 protein, which we have demonstrated to promote corneal healing without fibrosis, would exhibit safety and biocompatibility in vitro and in vivo. **Methods:** Hydrogel optimization was performed based on varying concentrations of poloxamer 407, poloxamer 188, and hydroxypropyl methylcellulose. Hydrogels were characterized and potential toxicity was evaluated in vitro in cultured corneal epithelium, fibroblasts, and endothelium. In vivo safety and tolerability were assessed in mice and hydrogels were used to evaluate corneal healing following alkali injury. **Results:** The optimized hydrogel formulation did not result in any detrimental changes to the corneal cells and released functional rhMG53 protein for at least 24 h. In vivo rhMG53-loaded hydrogels improved re-epithelialization, reduced stromal opacification and vascularization, and promoted corneal nerve density. Mechanistically, rhMG53 reduced vascular endothelial cell migration and tube formation by inhibiting pSTAT3 signaling. **Conclusions:** Taken together, our poloxamer-based thermoresponsive hydrogel effectively released rhMG53 protein and enhanced multiple corneal healing outcomes.

## 1. Introduction

Corneal wound healing is a complex and coordinated process, involving repair of the epithelial layer, migration of viable epithelial cells and fibroblasts for wound closure, and stimulation of cellular proliferation for tissue regeneration. Prevention of excessive stromal myofibroblast activation is also imperative to avoid fibrosis and opacification, which can compromise the transparency of the cornea [1]. The cornea is a unique structure that maintains its optical clarity by remaining completely avascular. Diseases in which the transparency of the cornea is compromised are the third most common cause of blindness worldwide [2,3]. Although the etiology of these diseases may be distinct, many converge on common pathways, leading to inflammation, loss of nerve function, and growth of vessels into the tissue, a process known as corneal vascularization [4,5,6]. An approach that can functionally target multiple steps in corneal wound healing may have the potential to significantly improve healing outcomes, leading to novel therapeutic options.

Mitsugumin 53 (MG53), a TRIM family protein, is an essential component of the cell membrane repair machinery, serving as a molecular bandage to facilitate repair of multi-tissue injuries [7,8]. MG53 protein is present in the human tear film, aqueous humor, and corneal epithelial cells, suggesting its potential function in corneal homeostasis and wound healing [9]. Our previous work demonstrates that, in vitro, the recombinant human (rh)MG53 protein protects against injury to human corneal epithelial cells (hCECs), can be taken up by corneal fibroblasts, and reduces pro-fibrotic signaling [9,10]. Following alkaline injuries to mouse and rat corneas, topical administration of rhMG53 protein facilitates corneal wound healing and improves corneal transparency by reducing stromal fibrosis and vascularization [9,10].

Several barriers exist when considering topical ocular drug delivery. The majority of the barrier function within the cornea exists within the epithelium, which contains tightly packed cells joined together by multiple junctional complexes at different depths [11,12,13]. Furthermore, eyelid movement and tear film kinetics frequently results in dispersion of topical medications into the nasolacrimal drainage system shortly after solutions are applied to the ocular surface [13,14]. Short residence time of protein therapeutics on the cornea is a major challenge to reducing scar formation. Due to these physiological barriers, the bioavailability of topical drugs is low. Conventional topical administration often requires higher dosages or repeated administration to stimulate a therapeutic effect, which lowers overall efficacy [15]. As observed in our prior work, healing outcomes improved with topical application of rhMG53; however, multiple treatments were required daily [9,10]. Clinically, there is an obvious need to enhance rhMG53 bioavailability to promote tissue residency time, thus simplifying dosing and improving patient compliance, while still achieving antifibrotic effects. Previous work has incorporated corneal treatments such as nerve growth factor, keratinocyte growth factor, verteporfin, or amniotic membrane proteins [16,17,18,19]; however, few studies have resulted in successfully minimizing application frequency while simultaneously improving multiple healing outcomes.

The goal of this study was to develop and characterize a thermosensitive hydrogel that could release physiologically relevant doses of rhMG53 to the ocular surface over an extended period of time to promote healing. Reducing the frequency of topical application was an additional goal, which would have the potential to improve patient compliance and reduce possible ocular irritation, complications that are frequently observed in people using topical therapeutics. We hypothesized that the Pluronic gels used here would remain at a sol state at room temperature while turning into a more viscoelastic gel state at physiological temperatures upon ocular application. This, in conjunction with hydroxypropyl methylcellulose (HPMC), would promote retention on the ocular surface to improve rhMG53 bioavailability. We further hypothesized that rhMG53 would remain functional following release from our hydrogel to reduce tissue fibrosis and vascularization. Here, we demonstrated that once-daily treatment with our rhMG53-loaded thermosensitive hydrogels can significantly improve corneal wound healing. This work represents a novel therapeutic option to improve corneal clarity, maintain corneal nerve density, and reduce corneal vascularization.

## 2. Materials and Methods

### 2.1. Materials

Poloxamer 407, Poloxamer 188, sodium chloride (NaCl), sodium bicarbonate (NaHCO_3_), calcium chloride dihydrate (CaCl_2_·2H_2_O), bovine serum albumin (BSA), HPMC, sodium hydroxide (NaOH), and phosphate buffered saline (PBS) were purchased from Sigma-Aldrich Inc. (St. Louis, MO, USA). The lactate dehydrogenase (LDH) assay was purchased from Abcam (Cambridge, UK), the LIVE/DEAD assay from Invitrogen (Waltham, MA, USA), and 660 nm Protein Assay from Pierce Biotechnology (Rockford, IL, USA). The MTT (3-[4,5-dimethylthiazol-2yl]-2,5-diphenyl-tetrazolium bromide) assay was purchased from R&D Systems (Minneapolis, MN, USA). For cell culture, KGM-2 culture media was purchased from Lonza (Walkersville, MD, USA), human corneal endothelial cell culture media from Celprogen (Torrance, CA, USA), Dulbecco’s modified Eagle’s media (DMEM), F-12K media, antibiotic, and fetal bovine serum (FBS) were purchased from Gibco (Carlsbad, CA, USA), endothelial cell growth supplement from Fisher Scientific (Waltham, MA, USA), and Matrigel from Corning Inc. (Corning, NY, USA). IL-1β was purchased from R&D Systems. Antibodies against STAT3, p-STAT3, and β-actin were purchased from Cell Signaling (Danvers, MA, USA), CD31 antibody was from BD Biosciences (Franklin Lakes, NJ, USA), βIII-tubulin from ThermoFisher, and the secondary antibodies and mounting media with DAPI from Invitrogen. All materials were used as received without any further modifications.

*E. coli* fermentation was used to obtain high-quality (>98% purity) rhMG53 protein as described [20]. The membrane protective activity of rhMG53 from each preparation was determined with established micro-glass bead injury assay as described previously [20,21].

### 2.2. Hydrogel Optimization

Hydrogel formulation optimization was performed based on varying Poloxamer 407 and HPMC concentrations using a 3^2^ factorial design. This experimental design can study the effect of a single factor and interactions between factors on the values of responses (dependent variables) and provide a more accurate regression equation than the 2^k^ model, due to more data presentation [22]. The concentration of Poloxamer 188 was held constant in all samples evaluated. The concentration of Poloxamer 407 (X_1_) and the concentration of HPMC (X_2_) were chosen as independent variables in 3^2^ full factorial designs, while drug release (Y_1_) and gelling temperature (Y_2_) were chosen as dependent variables [23,24]. Optimization with a three-level factorial resulted in ten variations of the formula (Supplemental Data, Table S1). After initial tests evaluating pH, gelling temperature, and adhesion, one formulation (Table 1) was determined to be the top candidate formation and subsequently characterized in more detail.

### 2.3. Preparation Methods

In situ poloxamer gel preparation was performed on ice, using cold PBS, and incubated at 4 °C [25,26]. Each reagent was sterilized in a UV chamber for 20 min prior to addition into the poloxamer gel. A measured quantity of Poloxamer 407 (1800 mg) was added to 60% of the desired volume (6.0 mL) of cold Dulbecco’s (D)PBS and stirred for 1 h. Poloxamer 188 (500 mg) was then added to the solution and stirred for 30 min; finally, HPMC (100 mg) was added and stirred for an additional 30 min. The total solution was kept at 4 °C overnight. Following overnight cold incubation, the remaining 40% of the desired volume of DPBS (4.0 mL) was added and stirred in ice for 1 h. Synthesized hydrogels were stored at 4 °C before use. Prior to in vitro or in vivo testing, 0.8 mg/mL rhMG53 (in PBS) was loaded into the hydrogels.

### 2.4. Characterization

#### 2.4.1. Clarity, Transmittance, and pH

The formulation pH was measured using a pH meter (Mettler–Toledo, Columbus, OH, USA) which was previously calibrated using standard buffers. The clarity was determined by using visual inspection against a black and white background. Transmittance at 450 nm was measured by calculating the percentage transmittance using a microplate reader (Thermo Fisher Scientific, Waltham, MA, USA), with distilled water as the blank [27]. The transmittance was calculated using the following equation: $Transmittance\left(\%\right)=10^{-\left({OD}_{sample}-{OD}_{blank}\right)}$. Six independent replicates were included. Light transmittance and pH were repeated, as described above, using hydrogels that were four weeks old. Hydrogels were stored at 4 °C after they were formulated and remained at 4 °C until their evaluation. Three replicates were used for each test.

#### 2.4.2. Gelling Temperature

Dynamic oscillatory rheology was performed for calculating the gelling temperature on a rheometer (Malvern Panalytical, Malvern, UK) equipped with a thermostat bath. All the samples were kept at 4 °C prior to evaluation. The gelling temperatures were determined at a fixed frequency of 0.1 Hz. The samples were heated at a rate of 1 °C/min from 20 to 45 °C. The gelation temperature was defined as the temperature where G′ (elastic modulus) crossed the G′′ (viscous modulus), which is the transition from sol to gel formulation. Rheology tests were repeated three times and the average values are reported as the gelling temperature.

#### 2.4.3. Frequency and Time Sweep Test

Frequency sweep tests were performed under physiological conditions (35 °C) and at room temperature (25 °C) using the oscillation frequency mode to investigate the effect of increasing the frequency from 0.1 to 10 Hz with a fixed rate of 1% strain, determined to be within the linear viscoelastic region, on the gel’s viscoelasticity properties. Time sweeps were also recorded at physiological temperature (35 °C) for 10 min and gelation time was determined by the time where crossover occurred between G′ and G″. All tests were performed three times.

#### 2.4.4. Hydrogel Adhesion

Hydrogel retention time is an important factor in the promotion of drug bioavailabilty. To minimize animal use, eggshells were used as a surrogate surface to initially evaluate the adhesive properties of the hydrogel, as has been published by others [28]. Acknowledging obvious differences between the cornea and the eggshell, there are similarities between the two structures including curvature and a high concentration of collagen type I, hyaluronic acid, keratin sulfate, and dermatan sulfate [29,30]. Eggshells were cleaned with soap and water for one minute and dried before being placed in a humidified incubator set to physiological conditions (35 °C). Eggshells were warmed for one hour before 50 μL of hydrogel was placed on the curved surface of the shell. Eggshells were placed in the incubator and rotated; hydrogel retention was photographed after initial application of the hydrogel and at 1, 4, and 8 h. Based on Design of Experiments (DoE) approach, we initially tested adhesion of formulation F6 (Table S1, Supplemental Data). Due to insufficient adhesion of the hydrogel to the eggshell (Figure S1, Supplemental Data), the amount of HPMC was increased (Table 1). The optimized gel formulation (ratio of Poloxamer 407:Poloxamar 188:HPMC was 18:5:1) was tested on the eggshell using freshly made hydrogels and using hydrogels that were four weeks old. Older hydrogels were stored at 4 °C after they were formulated and remained at 4 °C until their evaluation. Three replicates were used for each test.

### 2.5. Drug Release Assay

To investigate hydrogel drug release properties, the following protocol was performed, based on the literature, with the following modifications [28]. Blank hydrogel liquid precursors (sol) and rhMG53-loaded (0.8 mg/mL) sol were weighed into a pre-weighed microcentrifuge tube and incubated at 37 °C for 60 min to allow for gelation. An equal volume of artificial tear solution (0.67 g NaCl, 0.20 g NaHCO_3_, 0.008 g CaCl_2_·2H_2_O in 100 g distilled water) was then added to the microcentrifuge tube along the tube wall and incubated at 37 °C. All of the artificial tear solution was removed at different time intervals (0.5, 1, 2, 4, 6, and 24 h), fresh artificial tear solution was added, and the process continued. Quantification of protein released into the artificial tear solution was analyzed using the 660 nm Protein Assay, according to manufacturer’s instructions. All release tests were repeated four times.

### 2.6. Drug Release Kinetic Models

Three models were employed to examine the release kinetics, including Korsmeyer–Peppas, Higuchi, and first-order models [31]. The absorbance readings were converted to percentages of cumulative drug release before applying the drug release kinetic models.

The Korsmeyer–Peppas model is as follows: $f1=Mi/M\infty =Kt^{n}$ where f1 is the amount of drug released, M∞ denotes the amount of drug at the equilibrium state, Mi stands for the amount of drug released over time t, K represents the release velocity constant, and *n* is the exponent of release as the function of time t.

The Higuchi model is as follows: $f1=Q=K_{H}\sqrt{t}$ where $K_{H}$ is the release constant of the Higuchi model.

The first-order model is as follows: $\log Q_{1}=\log Q_{0}+k_{1}t/2.303$ where $Q_{1}$ is the amount of active agent released at time t, $Q_{0}$ denotes the initial amount of drug dissolved, and $k_{1}$ stands for the first-order constant.

### 2.7. Cytocompatibility and Bioactivity

The three main cellular elements of the normal cornea were used in these assessments. Immortalized human corneal epithelial cells (hCEC) were generously gifted by Dr. D. Robertson, University of Texas Southwestern Medical School and grown in KGM-2 medium [32]. Primary stromal fibroblast cultures were obtained by de-identified cadaveric globes from the National Disease Research Interchange (Philadelphia, PA, USA). Following receipt of globes, the corneal epithelium was removed, and a 50% depth keratectomy was made in the axial cornea to ensure isolation of corneal stroma. The excised stromal tissue was allowed to adhere to culture dishes prior to being covered with DMEM containing 1% antibiotics/antimycotics and 10% FBS. Immortalized human corneal endothelial cells (hCEndoC) were purchased from Accegen (Fairfield, NJ, USA) and grown in Human Corneal Endothelial Cell Culture Media. All cells were grown at 37 °C and 5% CO_2_, and growth medium was replenished every other day. Cells were allowed to grow to 90% confluence before passage; primary cells were passaged no more than five times.

To assess the cytocompatibility of the hydrogel, cells were seeded (0.5 × 10^5^ cells/well) on the bottom well of 24-well transwell plates and allowed to incubate overnight. The next day, 200 μL of hydrogel was added to the upper transwells and cells were incubated for another 24 h. A LDH assay was used to assess cell viability, each following manufacturer’s instructions. Viability assays were repeated six times.

Retained bioactivity of rhMG53 was assessed using primary human corneal fibroblasts. Cells were seeded (0.5 × 10^5^ cells/well) at the bottom well of 24-well transwell plates and allowed to incubate until confluence. Cells were treated with vehicle or 0.045 N NaOH in culture media for 60 s, followed by PBS washing (three times). Hydrogel (200 μL, with or without 0.8 mg/mL rhMG53 loaded) was added to the upper transwells and cells were incubated for another 24 h. LIVE/DEAD staining, following manufacturer’s recommendations, was used to assess cell viability. A representative image was taken at the centre of each well and ImageJ (version 1.54) was used to assess viable and non-viable cell numbers. Six replicates were included in each experiment.

To ensure that functional rhMG53 was released from the hydrogels after two weeks of storage, hydrogels were formulated and loaded with rhMG53, as described above, then stored at 4 °C until their evaluation. hCEC were seeded (0.5 × 10^5^ cells/well) on the bottom well of 24-well transwell plates and allowed to incubate overnight. The next day, 200 μL of hydrogel was added to the upper transwells and cells were incubated for another 24 h. A MTT assay was used to assess cell proliferation, following manufacturer’s instructions; four replicates were included in each experiment.

### 2.8. Vascular Endothelial Cells

Normal primary human vascular endothelial cells (HUVEC) were purchased from ATCC (Manassas, VA, USA) and grown in F-12K medium containing endothelial cell growth supplement. Cells were passaged no more than five times.

A LDH assay was used to assess cell viability; HUVEC were treated with rhMG53 (0, 10, 25, 50, or 100 µg/mL) for 24 h. Viability assays were repeated five times. To perform the scratch assay, HUVEC were seeded in (0.5 × 10^5^ cells/well) at the bottom well of 24-well plates, allowed to grow until 90% confluence, and scratched with a pipette tip. Cells were then washed with DPBS and treated with rhMG53 (0, 10, or 50 µg/mL) for 24 h. Photomicrographs were taken immediately after the scratch and after rhMG53 treatment; the change in scratch area was measured using ImageJ (version 1.54). Six replicates were included in each experiment.

To evaluate tube formation, HUVEC were seeded in (10^5^ cells/well) at the bottom well of 24-well plates that had first been coated with Matrigel. Cells were then incubated with VEGF-A (10 ng/mL), rhMG53 (50 µg/mL), or VEGF-A + rhMG53 for 18 h. Tube formation was photographed in five different locations within each well and analyzed using ImageJ [33]. For statistical analysis, four wells were seeded per condition.

### 2.9. Western Blotting

HUVEC were treated with IL-1β (10 ng/mL) ± rhMG53 (50 µg/mL) for 24 h before being lysed in ice-cold RIPA lysis buffer supplemented with protease and phosphatase inhibitor. Ten micrograms of protein were added to each lane and separated using SDS-PAGE, then transferred to PVDF membranes. Membranes were blocked and incubated with primary antibodies overnight (1:2000 STAT3, pSTAT3; 1:1000 β-actin) at 4 °C. Following extensive washing, membranes were incubated with species-specific secondary antibodies and imaged. Densitometry was determined using the BioRad ChemiDoc Imaging System (Hercules, CA, USA). Four replicates were included in each experiment.

### 2.10. In Vivo Testing

All animal care and usage followed the NIH and ARRIVE guidelines and were in accordance with the ARVO Statement for the Use of Animals in Ophthalmic and Vision Research. Rodent studies received IACUC approval from The Ohio State University (Protocol 2016A00000017). For all corneal wound healing models, injury was induced under anesthesia, and all animals received topical antibiotics as well as topical and systemic analgesics for at least 72 h for pain management. For all experiments, C57Bl/6J (Jackson Laboratory, Bar Harbor, ME, USA; both male and female, age of 3 months) were used. Sample size was determined by power analysis and based on our prior publications [9,10]. No animals or data points were excluded from analysis. In vivo images were captured using the Leica THUNDER Imaging System (Deerfield, IL, USA).

Tolerance and biocompatibility were first assessed using unloaded hydrogels. Five microliters of hydrogel was gently placed in the inferior conjunctival fornix daily for 14 days. Mice (*n* = 15/group) were assessed for conjunctival hyperemia, chemosis, ocular surface damage, corneal edema, and excessive lacrimation or pawing.

To determine the effectivity of rhMG53-loaded hydrogel as a treatment of corneal wounds, a wound was introduced by placing a 1.5 mm piece of filter paper soaked in 1 N NaOH on the axial cornea for 30 s. The corneas were then rinsed with 20 mL of saline; fluorescein stain was applied to the cornea to verify corneal ulceration. Animals were randomly assigned to a treatment group. Wounded corneas received topical hydrogel (unloaded or loaded with 0.8 mg/mL rhMG53; *n* = 7/group) once a day for 10 days. To reduce topical application time and maximum retention on the ocular surface, 5 µL of hydrogel was placed in the inferior conjunctival fornix. The size of the corneal wound was verified daily using fluorescein. In conjunction with the use of fluorescein dye to monitor healing rates, the following were clinically evaluated by a masked examiner: corneal opacification, vascularization, conjunctival edema, hyperemia, and discharge. A published scoring system was used to evaluate the eyes and adnexa; the corneal opacity scoring table is summarized in the Supplemental Data (Table S2) [34]. At 10 days following injury, animals were humanely euthanized, and globes were enucleated. In all subsequent histological analyses, the individual was masked to the treatment.

For flat mount staining, eyes were fixed in 1.5% PFA at room temperature (RT) for 1 h, then washed twice with PBS (5 min each). Corneas were then dissected, washed with PBS five times (5 min each wash), incubated in 1% Triton-X100 in PBS, and shaken for 60 min at RT. Tissue was then placed in blocking buffer (20% BSA, 0.3% Triton-X100, 0.1% Tween-20 in PBS) for 30 min at RT, before application of primary antibody (1:100 diluted in blocking buffer) and incubated at RT for 2 h, then transferred to 4 °C overnight. Subsequently, corneas were washed five times with washing buffer (0.1% Tween-20 in PBS) for 5 min each time at RT, and secondary antibodies (1:500 diluted in blocking buffer) were applied and incubated at RT for 2 h. Finally, the corneas were washed with washing buffer five times at room temperature for 10 min each time; four cuts were made in the corneas to facilitate mounting on slides.

The primary antibodies used for staining were anti-CD31 and anti-βIII-tubulin. Secondary antibodies were Alexa Fluor 488 Goat anti-Rabbit IgG and Alexa Fluor plus 647 Donkey anti-Rabbit IgG. The immunofluorescence staining images were captured using the ECLIPSE Ti2 microscope (Nikon; Melville, NY, USA). All image quantification (i.e., fluorescein, immunofluorescent staining) were quantified using ImageJ software (version 1.54) [35]. The neovascularization ratio was calculated by dividing the area occupied by blood vessels and the total corneal area. The nerve area ratio was calculated similarly by dividing the nerve-stained area by the total corneal area.

### 2.11. Statistical Analysis

Summary data are shown as mean ± standard deviation. Statistical analyses were conducted in GraphPad Prism v10.3.1 (Boston, MA, USA). Statistical analysis included Mann–Whitney and Kruskal–Wallis tests, as appropriate. *p* < 0.05 was considered statistically significant.

## 3. Results

### 3.1. Synthesis and Characterization of Poloxamer Hydrogels

**Figure 1:**
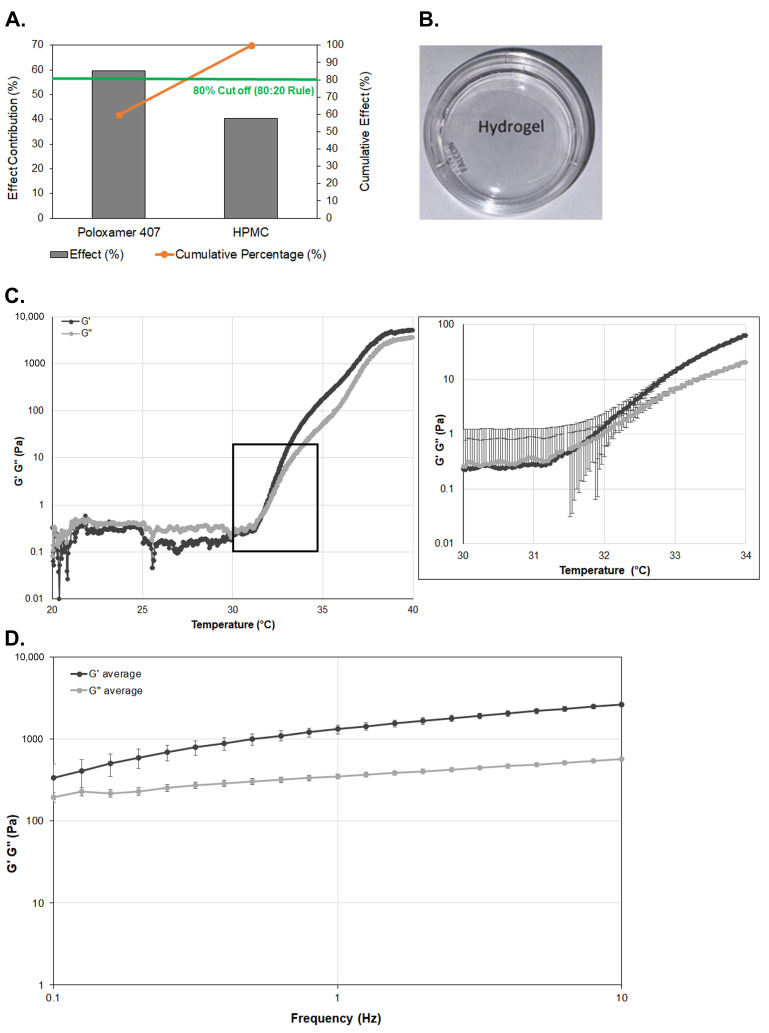
Transparency and physical characterization of the hydrogel. (**A**) The Pareto Plot, demonstrating the relative contributions of Poloxamer 407 and HPMC to the gelling temperature change. (**B**) Digital image of the hydrogel on a lettered background. Words are clearly visible through the transparent hydrogel-filled Petri dish. (**C**) Sol/gel transition of the thermoresponsive hydrogel. Black box in the left graph outlines the area that is magnified in the graph on the right, indicating the crossover point (*n* = 3). (**D**) Frequency sweep measurements of the hydrogel at different frequencies (0.1–10 Hz; *n* = 3).

Nine hydrogel formulations were initially prepared (Table S1, Supplementary Data), and using statistical experimental design, the optimal formulation which enabled gelation below the physiologically relevant temperature of the surface of the eye (35 °C) was determined and further characterized. The DoE approach was used to optimize the preparation of hydrogels and the Pareto Plot of this optimization, which indicates the importance of input factors, is shown in Figure 1A. The Pareto Plot, demonstrating the relative contributions of Poloxamer 407 and HPMC to the gelling temperature change, shows that based on the 80:20 Rule, Poloxamer 407 has the highest effect contribution at around 60%. This indicates that Poloxamer 407 plays the dominant role in influencing the gelling temperature. HPMC has a substantial but lesser impact than Poloxamer 407, contributing approximately 40%. Since Poloxamer 407 has a more substantial effect, focusing on its optimization concentration could be key to controlling the gelling temperature. Experimentally, based on initial hydrogel adhesion experiments, our DoE formulations lacked sufficient adhesion properties (Figure S1, Supplemental Data). Hydrogel adhesion was tested during the optimization process using eggshells. Based on initial characterization, formulation F6 (Table S1, Supplemental Data) was the most promising candidate; however, this formulation did not have sufficient adhesion to the eggshell (Figure S1, Supplemental Data). Accordingly, the amount of HPMC was increased to enhance the muscoadhesive properties (Table 1). The optimized gel formulation (ratio of Poloxamer 407:Poloxamar 188:HPMC was 18:5:1), both freshly made and hydrogel aged four weeks, maintained adhesion to the eggshell for 8 h (Figure S1, Supplemental Data).

The average pH of the optimized formulation was 7.2 ± 0.04 (*n* = 3), which is within the published acceptable range for the surface of the eye and was therefore not expected to cause irritation upon administration to the eye [22,25]. The pH of four-week-old hydrogel was 7.1 ± 0.02 (*n* = 3). The formulation was clear upon visual inspection (Figure 1B) and the transmittance in the visible light spectrum was 98.7 ± 0.2% (*n* = 3). Hydrogel that was aged for four weeks remained clear and the transmittance (98.2 ± 0.3%; *n* = 3) was not significantly different compared to fresh hydrogel.

For ease of application, the hydrogel needs to keep a sol state at room temperature while turning into a more viscoelastic gel state at physiological temperatures upon ocular application. The gelation temperatures of ophthalmic in situ gels are considered to be appropriate for ocular delivery if they are in the range of 25–34 °C [23,36]. If the gelling temperature is lower than 25 °C, a gel may form at room temperature, and if the gelling temperature is higher than 34 °C, the formulation may still stay at a sol state on the ocular surface, leading to excessive loss through rapid clearance from the surface of the eye. The gelling temperature and time for the optimized formulation as determined by rheological testing was 31.9 ± 0.4 °C and 85 s, respectively (*n* = 3; Figure 1C).

A frequency sweep test was additionally performed to analyze the viscoelastic properties of the formed gels at 35 °C, and the results are shown in Figure 1D (*n* = 3). At higher frequencies, the formulation had a G′ (storage modulus) notably higher than G′′ (loss modulus), indicating it behaved as a viscoelastic solid due to storage modulus being higher than loss modulus. At low frequencies, the difference between G′ and G′′ was lower, meaning the formulation behaved more like soft gel. This rheological behavior is expected for viscoelastic materials such as hydrogels because oscillation deformation applied at higher frequency leads to insufficient time for energy dissipation from the polymers, resulting in hydrogels acting more like solids with G′ being higher than G′′. On the contrary, oscillatory deformation applied at lower frequency leads to adequate time for energy dissipation, resulting in lower moduli [37]. The observed increased storage moduli, corresponding to greater elasticity of the in situ formulation, facilitates retention at the target site compared to a liquid, potentially providing prolonged drug release.

### 3.2. Functional rhMG53 Was Released over Time, with No Observed In Vitro Cytotoxicity

**Figure 2:**
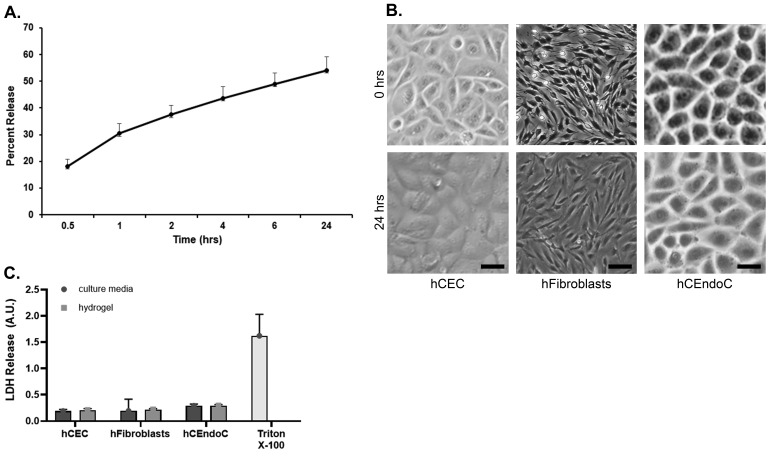
In vitro rhMG53 release and cell compatibility. (**A**) Quantification of rhMG53 release from hydrogels loaded with 0.8 mg/mL of rhMG53 after incubation in an artificial tear solution (*n* = 4). (**B**) Inverted microscopy images of cells (*n* = 6) grown in transwells with hydrogel at 0 and 24 h. No changes in cellular morphology or density were observed. Scale bar = 50 µm. (**C**) Cell viability, as measured using an LDH assay, demonstrates no cytotoxicity (*n* = 4); triton X-100 was used as a positive toxicity control.

On average, rhMG53-loaded hydrogels released 59.6 μg of protein over 24 h, equating to 54.1% of the loaded protein (*n* = 4; Figure 2A). The release profile showed a rapid burst release during the first 6 h, followed by a gradual release over time. The first-order model resulted in a K of 0.35 and R^2^ of 0.96, which was the best fit, indicating first-order release kinetics from these gels. The Higuchi model resulted in a K_H_ of 0.15 and R^2^ of 0.87, and the Korsmeyer–Peppas model resulted in a K_KP_ of 3.3, *n* of 0.27 and R^2^ of 0.84 (Figure S2, Table S3, Supplemental Data). These indicate Fickian diffusion of the protein from the hydrogels [31].

**Figure 3:**
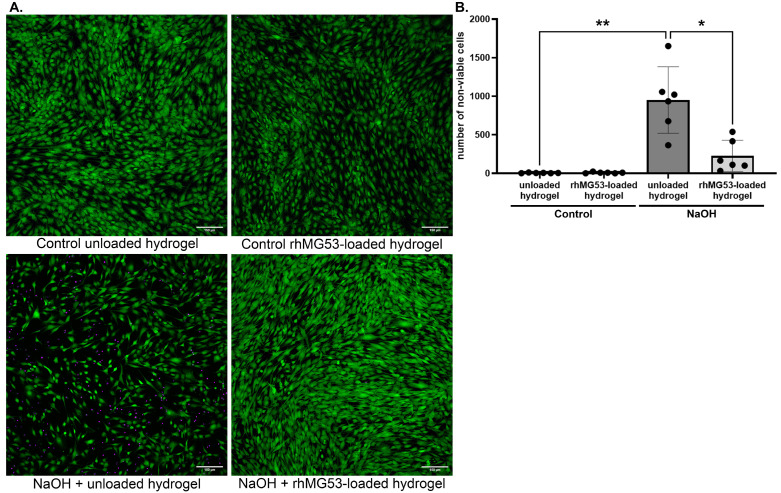
rhMG53 functionality when released from the hydrogel. Human corneal fibroblasts were treated with vehicle or NaOH for 60 s and allowed to recover in the presence of unloaded or rhMG53-loaded hydrogels for 24 h. (**A**) Live cells are labeled with Calcein-AM (green) while dead cells are labeled with EthD-1 (purple). (**B**) When quantified, NaOH significantly reduced (** *p* = 0.0139) cell viability while injured corneal fibroblasts treated with rhMG53-loaded hydrogels had significantly higher (* *p* = 0.0368) viability than unloaded hydrogels (*n* = 6). Scale bar = 150 µm. Statistical significance was assessed with the Kruskal–Wallis nonparametric test.

All cells grown in the presence of the unloaded hydrogels exhibited normal cellular morphology, maintained substrate attachment, and were grossly unchanged from controls (*n* = 6; Figure 2B). No significant cytotoxicity was observed, as assessed by LDH release assay (*n* = 4; Figure 2C). We have previously shown that rhMG53 can promote cell viability following injury [9]. To ensure that rhMG53 retained its functionality after being released from the hydrogels, human corneal fibroblasts were injured with NaOH and allowed to recover in the presence of unloaded or rhMG53-loaded hydrogels; viability was assessed using a LIVE/DEAD assay. As shown in Figure 3, NaOH significantly decreased (*p* = 0.0139) cell viability and rhMG53-loaded hydrogels significantly improved (*p* = 0.0368) cell viability following injury compared to unloaded hydrogels (*n* = 6). To ensure that functional rhMG53 was released two weeks after rhMG53 was loaded into the hydrogel, proliferation of hCEC was assessed. Two weeks was chosen because this is the expected duration of a corneal treatment regimen. Cells incubated in the presence of two-week-old rhMG53-loaded hydrogels had significantly (*p* < 0.001) increased proliferation compared to unloaded hydrogels (*n* = 4; Figure S3, Supplementary Data).

### 3.3. Topical Application of the Hydrogel Was Well-Tolerated and Promoted Corneal Healing In Vivo

**Figure 4:**
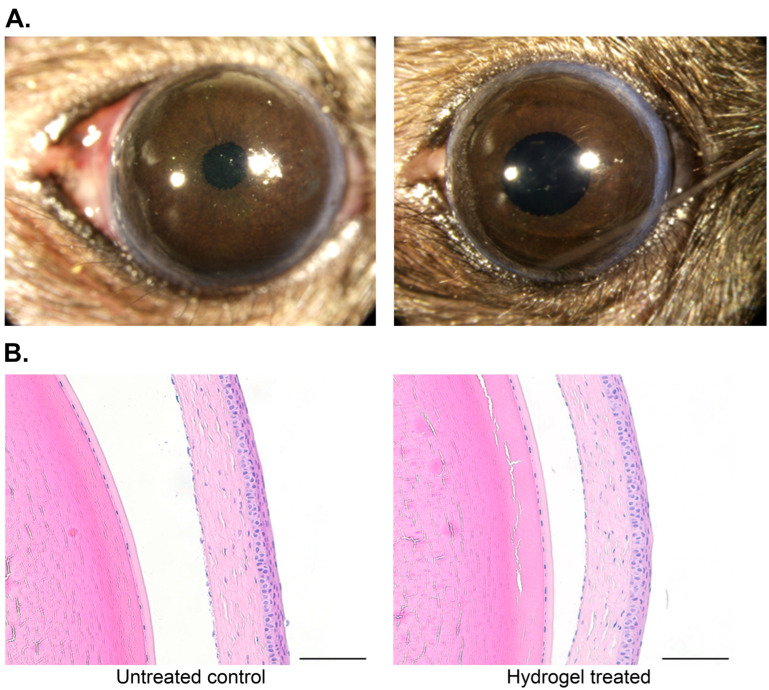
In vivo tolerability of the hydrogel. The hydrogel does not induce ocular surface irritation as observed (**A**) grossly and (**B**) histologically; all images are representative of animals on Day 14 (*n* = 15/group). Scale bar = 100 μm.

To confirm the biocompatibility of the hydrogels, mice were treated daily with topical hydrogel for two weeks. As shown in Figure 4A, treatment with the hydrogel resulted in no ocular changes, such as hyperemia, blepharospasm, or edema, and the mice displayed normal behavior, including normal blinking and no observable changes in nasolacrimal drainage. Note that any observed change in pupil diameter was the result of light duration and intensity (i.e., flash), and not related to treatment. Histological analysis further supported the tolerability of the hydrogel, in which no changes were noted in the corneal epithelial, stromal, or endothelial cell density (*n* = 15/group; Figure 4B).

**Figure 5:**
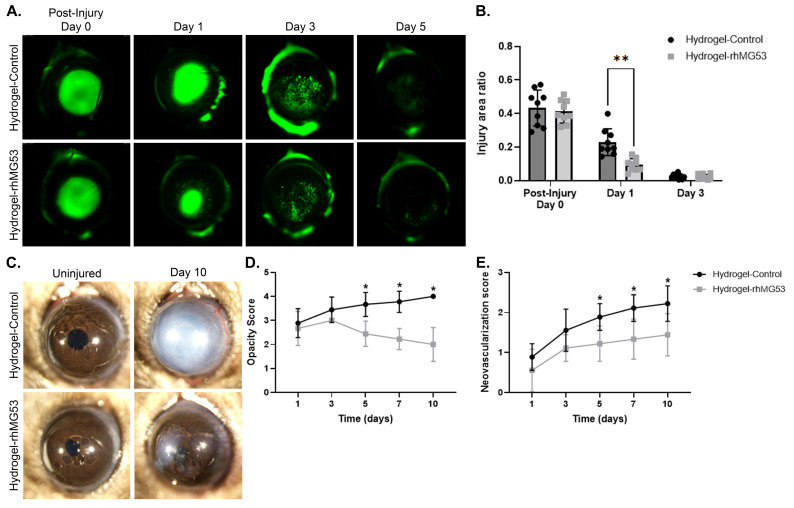
In vivo corneal healing in mice treated with hydrogel. (**A**) Representative photographs and (**B**) quantification of corneas after fluorescein staining immediately after injury and at designated post-injury time points. ** *p* = 0.002. (**C**) Representative photographs and (**D**) quantification of corneal stromal opacity during healing. * *p* < 0.0001. (**E**) Quantification of vascularization over time. * *p* < 0.001. *n* = 9/group. Statistical significance was assessed with the Mann–Whitney U nonparametric test.

**Figure 6:**
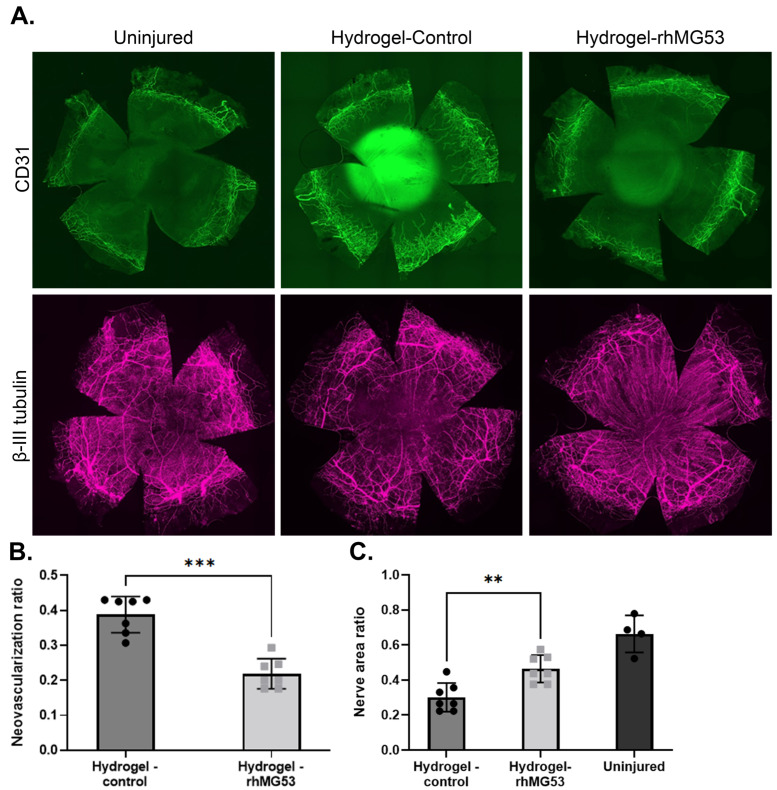
Flat mount staining of injured corneas following treatment for 10 days. (**A**) Staining against the vascularization marker CD31 and the neuronal marker β-III tubulin. Magnification 10×. Quantification of (**B**) CD31 (*** *p* = 0.0006) and (**C**) β-III tubulin (** *p* = 0.006) expression. Injured corneas treated with unloaded or rhMG53-loaded hydrogels (*n* = 7/group); uninjured corneas (*n* = 4). Statistical significance was assessed with the Mann–Whitney U nonparametric test.

We previously demonstrated that rhMG53 in an aqueous solution could improve corneal healing when applied topically multiple times a day [9,10]. One of the goals of this study was to reduce the frequency of topical applications required for effective rhMG53 treatment. In the current study, following alkali injury, topical application of unloaded or rhMG53-loaded hydrogels was given once a day in the conjunctival fornix (*n* = 9/group). Corneal re-epithelialization, as assessed by fluorescein staining, was significantly improved (*p* = 0.002) on the first day of healing in the rhMG53-loaded hydrogel group compared to control hydrogel group (Figure 5A,B). No differences in epithelial healing were observed beyond day 3 post-injury, and wound re-epithelialization was completed by day 5 in both groups. Opacity scores were significantly reduced (*p* < 0.0001) in corneas receiving rhMG53-loaded hydrogels compared to unloaded control hydrogels on days 5, 7, and 10 post-injury (Figure 5C,D). rhMG53-loaded hydrogels significantly reduced (*p* < 0.001) corneal vascularization scores when compared to controls (Figure 5E). Flat mount staining revealed that corneas treated with rhMG53-loaded hydrogels had significantly less (*p* = 0.006) CD31 staining (*n* = 7; Figure 6A,B) than those treated with unloaded hydrogels, confirming that rhMG53 inhibited corneal vascularization. In addition to reducing corneal vascularization, flat mount staining for the neuronal marker β-III tubulin demonstrated significant improvement (*p* = 0.006) in nerve density in injured corneas treated with rhMG53-loaded hydrogels compared to unloaded hydrogel controls (*n* = 7; Figure 6A,C).

**Figure 7:**
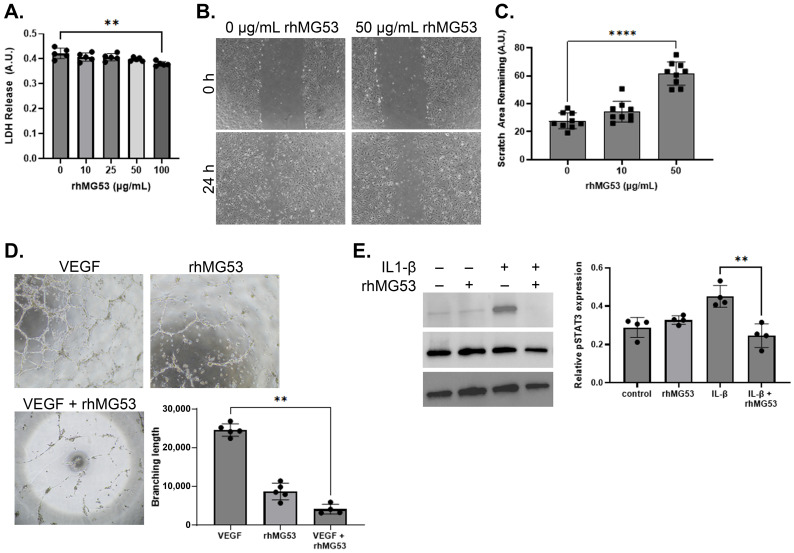
rhMG53 inhibits vascular endothelial cell migration and tube formation. (**A**) rhMG53 did not induce cellular toxicity in HUVEC up to 100 μg/mL (*n* = 5; ** *p* = 0.01). (**B**) Representative photomicrographs 24 h after scratch wounding, HUVEC treated with 50 μg/mL rhMG53 had significantly less migration and proliferation compared to control (*n* = 9). Magnification 4×. (**C**) Quantification of the scratch area remaining at 24 h (**** *p* < 0.0001). (**D**) Representative photomicrographs and quantification of tube formation when HUVEC was seeded on Matrigel, followed by treatment with VEGF (10 ng/mL) with or without rhMG53 (50 μg/mL) for 18 h. rhMG53 significantly reduced tube length in VEGF-treated HUVEC (*n* = 4; ** *p* = 0.0029). Magnification 4×. (**E**) HUVEC treated with IL-1B (10 ng/mL) with or without rhMG53 have decreased pSTAT3 protein expression; when normalized to total STAT3 expression, rhMG53 significantly (** *p* = 0.0084) decreased pSTAT3 (*n* = 4). Statistical significance was assessed with the Kruskal–Wallis nonparametric test.

Given the observed reduction in corneal angiogenesis, we further evaluated the effect of rhMG53 on HUVEC. To evaluate potential cytotoxicity, HUVECs were treated with various concentrations of rhMG53, and only the highest dose (100 µg/mL) resulted in a significant decrease in cell viability (*n* = 5; Figure 7A). rhMG53 treatment significantly inhibited both vascular endothelial cell migration (*p* < 0.0001, *n* = 9) and tube formation (*p* = 0.0029, *n* = 4; Figure 7B–D). Moreover, in HUVECs treated with IL-1β, rhMG53 significantly reduced (*p* = 0.0084) IL-1β-induced increase in p-STAT3 expression (Figure 7E; *n* = 4). These findings align with previous research indicating that IL-1β and STAT3 signaling promote corneal vascularization [38,39,40].

## 4. Discussion

Maintaining corneal transparency is critical for preserving vision. Although corneal wounds can be treated with topical antibiotics, tear substitutes, and contact lenses, many of these traditional treatments only address one aspect of corneal healing and often fail to target the entire healing process. Even advanced corneal treatment strategies, such as growth factor-derived products or autologous serum, have shown limited clinical success, especially in reducing fibrosis and angiogenesis [41,42]. Application of proteins known to promote corneal health, such as nerve growth factor or amniotic membrane proteins [16,17,18,19], in topical hydrogels have not been successful in minimizing application time to once a day while still achieving significantly enhanced corneal healing via multiple mechanisms. In this study, we provided evidence that MG53 is multi-functional within the cornea to improve multiple healing outcomes, following daily topical administration.

In our previous work, we demonstrated enhanced corneal healing when rhMG53 treatment, delivered in an aqueous solution, was applied topically multiple times a day [9,10]. In order to reduce the frequency of application, increase corneal retention time, and achieve sustained rhMG53 delivery to the cornea, we developed a topical thermosensitive in situ gelling hydrogel. This hydrogel successfully released rhMG53 for at least 24 h. Although rhMG53 protein was retained within the hydrogel after 24 h, the study goal was to release protein at a dose sufficient to elicit a therapeutic response to the ocular surface over time. Based on our previous work, a minimum of 10 μg of protein is necessary to attain therapeutic efficacy, [9,10] which was successfully achieved with our hydrogel. Future studies should focus on refining the optimal rhMG53 dose released while minimizing the amount of protein retained within the hydrogel to reduce protein waste.

We observed a burst release that led to a higher initial rhMG53 dose, which may be advantageous to support initial tissue regeneration following wounding [43]. We have previously shown that rhMG53 can be taken up by corneal fibroblasts within one hour of application [9]; similar uptake rates have been observed by limbal epithelial cells (unpublished data). This burst release may promote the immediate anti-fibrotic and anti-angiotic signaling in the early stages of healing to promote tissue transparency. Alternatively, it is also possible that the observed burst release may reduce the effectiveness of the desired extended action of our hydrogel. Additional work is needed to ensure that a burst release is not detrimental to the effectivity of our hydrogel. Most drug delivery formulations have some form of burst release [43] and compared to conventional eye drops, other topical hydrogels, or soaked contact lenses, our formulation’s burst release is similar or relatively low [18,44,45,46,47]. Future formulations could focus on both reducing burst release and extending delivery, which may be achieved by applying these formulations as punctal plugs or fornix inserts, rather than topical eye drops.

Various hydrogels have been developed for ophthalmic drug delivery, including those that are sensitive to temperature, pH, or ions [48,49]. These hydrogels remain in solution until application to the target site, where the sol–gel transformation occurs in response to the appropriate stimuli, making in situ gels particularly unique. Thermosensitive and thermoreversible hydrogels using methylcelluolose, Pluronic, sodium hyaluronate, etc., are advantageous because the change in physical state is rapid and harmless to the native tissue [17,50]. In the current study, all components were selected due to their regulatory approval status [51,52], with the intent of facilitating rapid clinical translation. This reduces possible regulatory barriers when considering large-scale clinical application. That said, production scalability and product cost may still prove challenging when considering future clinical use.

Several hydrogel designs were considered for sustained release of rhMG53 [53], but these initial studies focused on designing a hydrogel delivery system with higher potential of clinical translation. In addition to being on the Generally Recognized as Safe (GRAS) list in the United States, poloxamers and HPMC were selected for use in our formulation due to the thermoresponsive properties of the poloxamers and the mucoadhesive properties of the HPMC [54,55]. Supporting the hydrogel composition, Poloxamer 407 was selected due to its sol–gel transition from liquid at room temperature to solid at body temperature and its non-ionic properties [56]. Furthermore, Poloxamer 407 has been demonstrated to increase stability of proteins [57]. Poloxamer 188 was used to modulate gelation temperature to be closer to the temperature of the ocular surface [58]. This enabled modulation of the ratios of the poloxamers to fine-tune the gelation temperature to be close to, but below, the temperature of the ocular surface. Several of the formulations in our preliminary investigations were eliminated from further evaluation due to a gelation temperature being too close to physiological temperature or a gelation temperature too close to room temperature. Finally, HPMC was added to enhance the viscosity and increase the contact time of the formulation on the ocular surface while it gelled, increasing bioavailability [58,59]. Overall, the developed hydrogels were easily administered and sustained release of bioactive protein at physiologically relevant levels. The generation of our hydrogel has high clinical translation due to the innovative incorporation of rhMG53 into its formulation, which would allow sustained release of the protein to facilitate corneal healing by reducing fibrosis and vascularization, while promoting tissue nerve growth.

Corneal vascularization, a sight-threatening condition affecting more than 1.4 million people per year [60], is characterized by the abnormal growth of blood vessels into the normally avascular cornea. This process is accompanied by inflammation, tissue scarring, and edema, all of which severely impairs vision [61]. Currently, there is no definitive, successful therapy for corneal vascularization, underscoring the need to identify alternative and effective treatments. Our data indicate that rhMG53 could serve as a safe and efficacious treatment for corneal angiogenesis. In vitro studies showed that rhMG53 reduced migration and tube formation in HUVECs. Within the cornea, cytokines, such as IL-1β, have been shown to promote corneal vascularization, and when IL-1β is neutralized, corneal angiogenesis is inhibited [38]. Mechanistically, we demonstrate that IL-1β-treated HUVEC had increased p-STAT3 expression, which is known to be activated in corneal angiogenesis following alkali injury [40,62,63]; concurrent treatment with rhMG53 significantly decreased p-STAT3 expression. Other studies have also demonstrated that rhMG53 can be internalized by HUVEC, reducing endothelial cell migration and inhibiting the Akt pathway, which interacts with p-STAT3 signaling [64,65]. Furthermore, TGF-β signaling promotes corneal fibrosis by regulating STAT3 signaling and we have previously demonstrated that rhMG53 can inhibit pro-fibrotic canonical TGF-β signaling in corneal fibroblasts [9,66,67]. These findings collectively support the multi-functional role of MG53 in the cornea, particularly in reducing vascularization. Future research should explore additional pathways and mechanisms, particularly signaling pathways linking inflammation and angiogenesis in the cornea.

The cornea is one of the most densely innervated tissues in the body, making corneal wounds exceptionally painful. Restoring corneal nerve density can not only promote patient comfort but also improve corneal healing outcomes [68]. Corneal sensory nerves play a critical trophic role for the epithelium and secrete multiple neuropeptides, such as calcitonin gene-related peptide and substance P [68,69,70], which are essential for corneal healing. The loss of corneal nerves abrogates the vital trophic role these nerves play [71,72]. We demonstrated that rhMG53 treatment significantly ameliorated NaOH-induced nerve regression in the cornea. Further research is needed to determine whether rhMG53 actively promotes nerve regrowth or simply protects existing nerves. Such experiments will clarify the molecular action of MG53 on nerve cells, which we hypothesized to have contributed to the enhanced corneal healing observed with rhMG53-loaded hydrogels.

Mechanistically, we have previously demonstrated that rhMG53 promotes tissue transparency by improving corneal epithelial viability and proliferation as well as reducing stromal fibrosis and vascularization [9,10]. Corneal fibroblasts were able to take up rhMG53 from the extracellular environment within one hour of treatment [9]; preliminary studies indicate limbal epithelium and bone marrow-derived stem cells can similarly take up rhMG53 (unpublished data). Ongoing studies in our laboratory seek to further clarify the mechanisms associated with this protein uptake by evaluating putative receptors. Furthermore, we are actively investigating modulation of the inflammatory response within the cornea following treatment with our rhMG53-loaded hydrogels.

It is important to note the limitations of the current study. Although we demonstrated that the hydrogel is stable at 4 °C for one month, similar to other formulations [73,74], stability beyond one month should be determined in future studies. We also demonstrated that the hydrogel is capable of releasing functional protein for two weeks, which is the expected treatment duration for many corneal wounds. Although rhMG53 protein exhibits stability in the lyophilized form and when resuspended into solution for experimental use [75,76,77], evaluating and improving extended release of rhMG53 may improve clinical application. While eye drops have several limitations, primarily rapid clearance from the ocular surface, they remain the most commonly used method for ocular drug delivery. In our current hydrogel formulation, we were able to fine-tune the gelation temperature and HPMC enabled the sol formulation to adhere to the ocular surface while it gelled, further reducing rapid clearance associated with most liquid formulations, such as the saline used in our previously published studies [9]. Future studies should quantify retention time of the hydrogel on the ocular surface, as well as work to improve ocular residency time and minimize burst release even further by investigating nanocomposites or encapsulation of rhMG53 in particles before loading in the gel, as has been demonstrated by others, for topical application [78,79]. Additional work should also determine diffusion and tissue retention of rhMG53, using both ex vivo and in vivo models, in an effort to more properly estimate rhMG53’s permeation profile.

## 5. Conclusions

In summary, we have developed a biocompatible, thermosensitive hydrogel capable of extended rhMG53 release to the ocular surface while maintaining protein functionality. This hydrogel significantly improved corneal healing following alkali injury when applied once daily in the conjunctival fornix. rhMG53 effectively mitigated various detrimental outcomes associated with stromal injury, including opacification, vascularization, and loss of corneal nerves. These findings further confirm the multi-functional role of MG53 in promoting corneal transparency and enhancing healing.

## Figures and Tables

**Table 1:** Optimized gel formulation.

Chemical	Formulation of In Situ Thermo-Responsive Gel (*w*/*v*%)
Poloxamer 407	18.0
Poloxamer 188	5.0
HPMC	1.0

## Data Availability

The original contributions presented in this study are included in the article/supplementary material. Further inquiries can be directed to the corresponding authors.

## Supplementary Materials

The following supporting information can be downloaded at: https://www.mdpi.com/article/10.3390/pharmaceutics17040526/s1, Table S1: Formulations of the unused gels, all made in DPBS; Table S2: Corneal opacity score system [34]; Figure S1: Hydrogel adhesion to a curved surface over time; Figure S2: Mathematical models of release data including (A) First order, (B) Higuchi, (C) Korsmeyer-Peppas; Table S3: Constants derived from Figure S2; Figure S3: In vitro release from hydrogels loaded with rhMG53 two weeks prior.
