# Supplementary material for: Development of an Ophthalmic Hydrogel to Deliver MG53 and Promote Corneal Wound Healing
Source: Pharmaceutics. 2025 Apr 16;17(4):526. doi: 10.3390/pharmaceutics17040526 (PMC12030682; doi:10.3390/pharmaceutics17040526)
Supplement: Supplementary file 1 [file pharmaceutics-17-00526-s001.zip › pharmaceutics-3529210-supplementary.pdf]

**Supplemental Data**

**Table S1:** Formulations of the unused gels, all made in DPBS.

| **Chemical** | **Formulation of in situ thermo-responsive gel (w/v%)** | | | | | | | | |
|---|---|---|---|---|---|---|---|---|---|
| | F1 | F2 | F3 | F4 | F5 | F6 | F7 | F8 | F9 |
| Poloxamer 407 | 17 | 17 | 17 | 18 | 18 | 18 | 20 | 20 | 20 |
| Poloxamer 188 | 5 | 5 | 5 | 5 | 5 | 5 | 5 | 5 | 5 |
| HPMC | 0.3 | 0.4 | 0.5 | 0.3 | 0.4 | 0.5 | 0.3 | 0.4 | 0.5 |

**Table S2:** Corneal opacity score system [35].

| Corneal opacity – severity | |
|---|---|
| 0 | Normal cornea. Appears with the slit lamp as having a bright gray line on the epithelial surface and a bright gray line on the endothelial surface with a marble-like gray appearance of the stroma. |
| 1 | Minimal loss of corneal transparency. With diffuse illumination, the underlying anterior segment structures are clearly visible, although corneal opacity is apparent to an experienced observer. |
| 2 | Mild loss of corneal transparency. With diffuse illumination, the underlying anterior segment structures are visible, although there is a reduction in the ability to appreciate their detail. |
| 3 | Moderate loss of corneal transparency. With diffuse illumination, there is a greater inability to see the details of the underlying anterior segment structures than with a score of 2, but the observer is still able to score aqueous flare, iris vessel congestion, observe for pupillary response, and note lenticular changes. |
| 4 | Severe loss of corneal transparency. With diffuse illumination, the underlying anterior segment structures cannot be seen so that the evaluation of aqueous flare, iris vessel congestion, pupillary response, and lenticular changes is not possible. |
| Corneal opacity – area | |
| 0 | Normal cornea with no area of corneal opacity. |
| 1 | 1% to 25% area of corneal opacity. |
| 2 | 26% to 50% area of corneal opacity. |
| 3 | 51% to 75% area of corneal opacity. |
| 4 | 76% to 100% area of corneal opacity. |

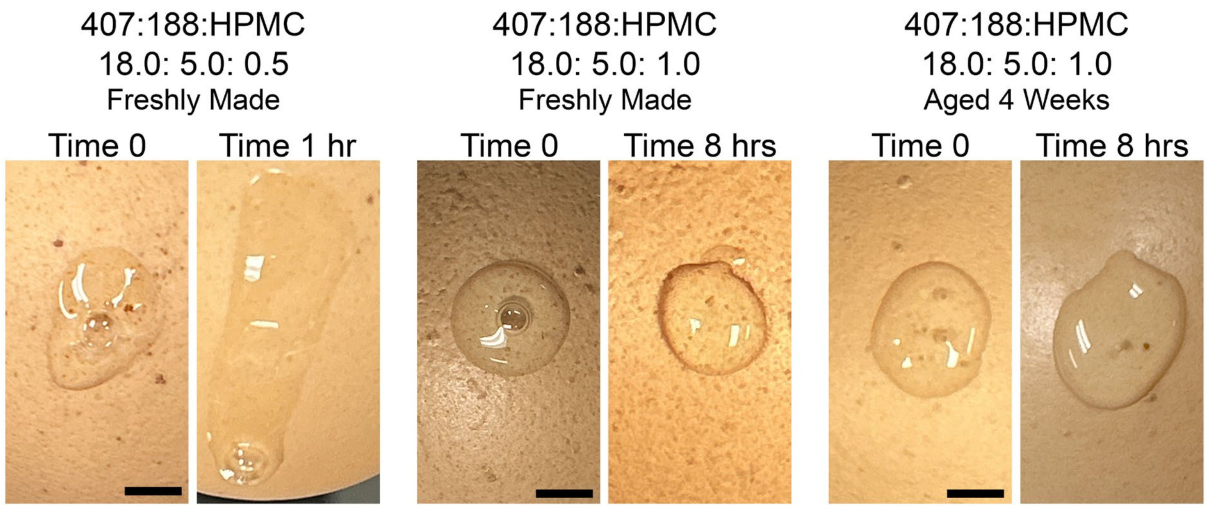


**Figure S1: Hydrogel adhesion to a curved surface over time.** Two formulations were tested on the curved surface of an eggshell to evaluate hydrogel adhesion over time. The ratio of Poloamer 407: Poloxomer 188: HPMC (w/v%) was either 18.0: 5.0: 0.5 (Formulation F6 in Table 1, Supplemental Data) or 18.0: 5.0: 1.0 (optimized formulation). Formulation F6 did not exhibit sufficient mucoadhesive properties and slide off the curved surface within 1 hour. The optimized formulation, fresh and aged, remained adhered to the curved surface for at least 8 hours. Scale bar = 5mm.

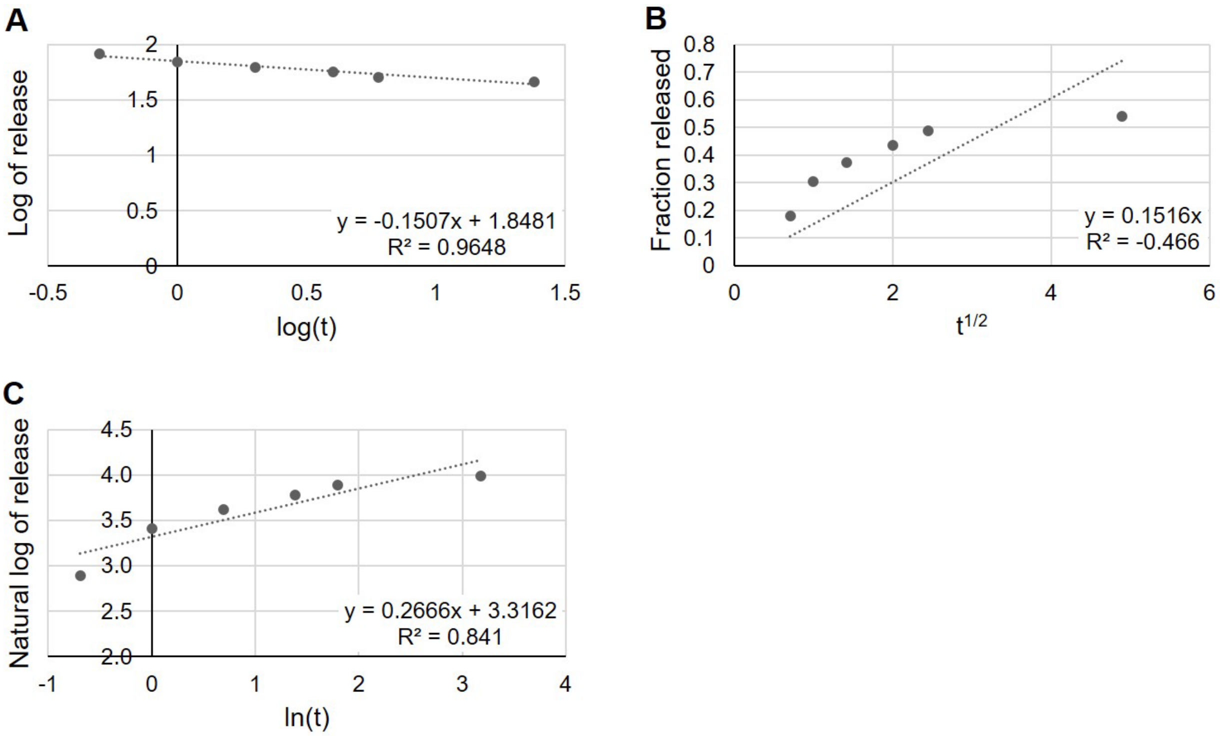


**Figure S2: Mathematical models of release data** including **A)** First order, **B)** Higuchi, **C)** Korsmeyer-Peppas.

**Table S3:** Constants derived from Figure S2.

| First Order | | Higuchi | | Korsmeyer-Peppas | | |
|---|---|---|---|---|---|---|
| K | $R^2$ | KH | $R^2$ | n | KKP | $R^2$ |
| 0.35 | 0.96 | 0.15 | 0.87 | 0.27 | 3.3 | 0.84 |

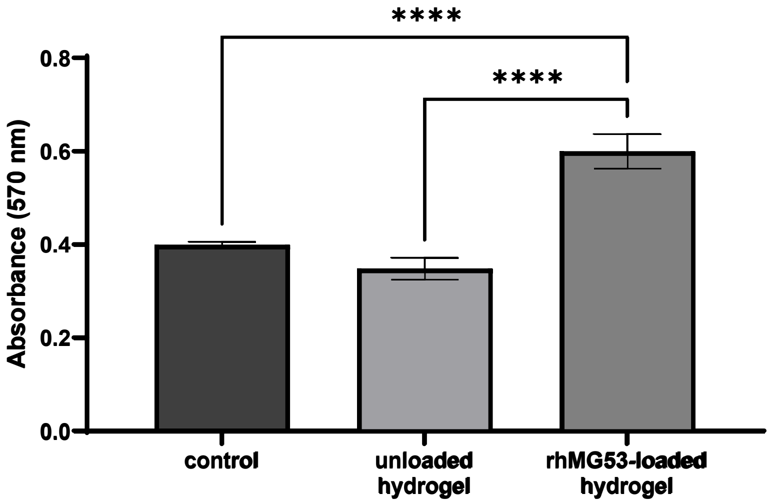


**Figure S3: In vitro release from hydrogels loaded with rhMG53 two weeks prior.** hCEC were treated with culture media, unloaded hydrogel (2-weeks old), or hydrogel loaded with rhMG53 2-weeks prior. rhMG53-loaded hydrogels significantly (**** $p<0.001$) increased proliferation (n=4). Statistical significance was assessed with the Kruskal-Wallis nonparametric test.
